# Mediterranean diet or extended fasting's influence on changing the intestinal microflora, immunoglobulin A secretion and clinical outcome in patients with rheumatoid arthritis and fibromyalgia: an observational study

**DOI:** 10.1186/1472-6882-5-22

**Published:** 2005-12-22

**Authors:** Andreas Michalsen, Markus Riegert, Rainer Lüdtke, Marcus Bäcker, Jost Langhorst, Myriam Schwickert, Gustav J Dobos

**Affiliations:** 1 Kliniken Essen-Mitte, Department of Internal and Integrative Medicine, Chair of Complementary Medicine at the University Duisburg-Essen, am Deimelsberg 34a, 45276 Essen, Germany; 2 Karl und Veronica Carstens Foundation, Essen, am Deimelsberg 34a, 45276 Essen, Germany

## Abstract

**Background:**

Alterations in the intestinal bacterial flora are believed to be contributing factors to many chronic inflammatory and degenerative diseases including rheumatic diseases. While microbiological fecal culture analysis is now increasingly used, little is known about the relationship of changes in intestinal flora, dietary patterns and clinical outcome in specific diseases. To clarify the role of microbiological culture analysis we aimed to evaluate whether in patients with rheumatoid arthritis (RA) or fibromyalgia (FM) a Mediterranean diet or an 8-day fasting period are associated with changes in fecal flora and whether changes in fecal flora are associated with clinical outcome.

**Methods:**

During a two-months-period 51 consecutive patients from an Integrative Medicine hospital department with an established diagnosis of RA (n = 16) or FM (n = 35) were included in the study. According to predefined clinical criteria and the subjects' choice the patients received a mostly vegetarian Mediterranean diet (n = 21; mean age 50.9 +/-13.3 y) or participated in an intermittent modified 8-day fasting therapy (n = 30; mean age 53.7 +/- 9.4 y). Quantitative aerob and anaerob bacterial flora, stool pH and concentrations of secretory immunoglobulin A (sIgA) were analysed from stool samples at the beginning, at the end of the 2-week hospital stay and at a 3-months follow-up. Clinical outcome was assessed with the DAS 28 for RA patients and with a disease severity rating scale in FM patients.

**Results:**

We found no significant changes in the fecal bacterial counts following the two dietary interventions within and between groups, nor were significant differences found in the analysis of sIgA and stool ph. Clinical improvement at the end of the hospital stay tended to be greater in fasting vs. non-fasting patients with RA (p = 0.09). Clinical outcome was not related to alterations in the intestinal flora.

**Conclusion:**

Neither Mediterranean diet nor fasting treatments affect the microbiologically assessed intestinal flora and sIgA levels in patients with RA and FM. The impact of dietary interventions on the human intestinal flora and the role of the fecal flora in rheumatic diseases have to be clarified with newer molecular analysis techniques. The potential benefit of fasting treatment in RA and FM should be further tested in randomised trials.

## Background

The colon is the home for a complex mixture of microorganisms that is critical for human health. The actual number of species that may be present is controversial; it has been estimated that >500 species coexist in the human colon [1]. It is believed that microflora contributes to the host's health by improving the intestinal tract's microbial balance [2]. The idea of purported health benefits from probiotic nutrients goes back to Elie Metchnikoff who suggested that Bulgarian peasants lived longer because of their yoghurt consumption which improves health by detoxifying putrefactive substances [3]. In the meantime a variety of potential mechanisms have been suggested to mediate the proposed associations between microflora and human health [4]. In the recent past interest in the potential of improving human health through modifications of the intestinal microflora has reemerged. Various dairy products and probiotic supplements claim such effects. While studies are limited, it appears that overall dietary patterns and the intake of various nutrients primarily affect general patterns of the fecal microflora [5-7]. At the same time, for subjects in feeding studies the same diet may have very different effects on the microflora, possibly due to differential effects of the diet on the individuals' underlying microflora composition, and/or underlying genetic differences [8]. Finally, experimental and observational data suggest that not only diet but also fasting and food restriction affect the intestinal microflora [9,10].

Unfortunately, studies that attempt to associate dietary interventions with changes in the microflora and disease outcomes are virtually nonexistent. Nevertheless, alterations in the fecal flora are increasingly believed to be contributing factors to many chronic inflammatory diseases and have attracted growing interest in the field of Complementary and Alternative Medicine [11]. First clinical evidence for the health-promoting effects of probiotic drugs or diets was derived from studies on inflammatory bowel disease and rheumatoid arthritis (RA). In RA, the beneficial effect of fasting followed by vegetarian diet on the clinical course of the disease has been demonstrated in randomised trials [12] and furthermore been linked to alterations of the fecal flora, as assessed by gas-liquid chromatography of bacterial cellular fatty acids [13]. Preliminary data indicate an increase in secretory immunoglobulin A (sIgA) concentrations following an extended period of fasting in patients with chronic pain syndromes and fibromyalgia (FM) and claimed to reflect improvements of immune function through these treatments [14].

Against this background we analysed the fecal flora with classical microbiological culture technique and evaluated the clinical outcome in a consecutive series of patients with RA or FM that underwent a 2-week inpatient Integrative treatment period with focus on nutritional therapies. We aimed to evaluate whether a) a Mediterranean diet or an intermittent 8-day fasting period are associated with changes in fecal flora, and, b) putative changes in fecal flora are associated with clinical improvements in the two selected diseases. Therefore, for all patients we analysed the quantitative aerob and anaerob bacterial flora, pH and concentrations of sIgA from stool samples at the beginning, at the end of the hospital stay and at a three months follow-up and related it to the nutritional intervention and the clinical outcomes.

## Methods

### Subjects

Study subjects were inpatients from an Internal and Integrative Medicine hospital department specialised in therapeutic lifestyle modification and nutritional therapies in internal diseases and rheumatic and orthopedic pain syndromes. The primary established diagnosis for all participants was RA or primary FM. The study sample consisted of consecutively admitted inpatients during two months which regularly stayed 14 days in the clinic. All patients were initially informed about the aim of the study and, according to the standard clinical treatment protocols, were offered a mostly vegetarian modified whole grain Mediterranean diet or participated in a supervised modified fast of eight days with 2 additional pre- and 3 post fast diet days. Patients were carefully instructed that the main purpose of fasting therapy was not weight reduction. Concomitant treatments included exercise, physical therapies, a structured program of stress reduction and some complementary therapies as massage and hydrotherapy. No supplements were given. According to a standardised protocol all patients received a similar amount of these adjunctive therapies. Exclusion criteria to the study included malnutrition, psychiatric disease, severe renal or hepatic insufficiency, unstable coronary artery disease, manifest endocrine disease, heart failure, history of cancer and treatment with methotrexat, azathioprin, high dose corticosteroids or antibiotics within the last 3 months. Further exclusion criteria to fasting consisted of manifest or prior eating disorder and severe obesity with a BMI > 40 kg/m^2^. Patients should not have followed any kind of specific diet in the preceding 3 months, as assessed by interview and a retrospective food frequency questionnaire. 51 of 58 screened patients volunteered to participate in the study. All patients gave their written informed consent to the study. The study protocol was approved by the local Ethics Committee.

### Protocol and dietary interventions

The observation period started at admission (study day 1), the dietary intervention started on the second clinical day (study day 2) with the first of two pre-fast days for fasting participants. At these pre-fast days fasting subjects received a 3350 kj (800 kcal)/day low-calorie and low-salt diet with intake of pure cooked rice and vegetables. Subjects then fasted from the evening of study day 3 to noon at study day 11, followed by two low-calorie diet days with stepwise reintroduction of foods. Normocaloric diet was reached again with the evening of study day 13 for fasting participants. During the 8-day modified fasting period the patients received free amounts of tea (no black or green tea), 200 cl fruit juice and small standardised quantities of light vegetable soup with a total maximum energy intake of 1255 kj (300 kcal)/day. Fasting started with the oral ingestion of a laxative salt (30–40 mg Glauber's salt). Patients were strongly advised to drink 2 to 3 l of fluids daily.

Patients in the diet group received a normocaloric Mediterranean diet, as adapted from the Lyon Diet Heart trial [15] and corresponding to an intake of 8372 kj (2000 kcal)/day. In brief, the diet consisted of 7 portions of fruit and vegetables daily, the abundant intake of whole grain bread, pasta and rice, two portions of fish/week and the exclusive use of olive oil and canola oil for all meal preparations. For all participants, fasting and non fasting-patients, caffeine-containing or alcoholic beverages were not allowed throughout the inpatient treatment. A dietician, trained nursing staff and a trained physician supervised the fasting and the control diet. All patients were recommended to follow a Mediterranean diet with a maximum of one to two portions meat/week after discharge from hospital. Throughout the inpatient period all participants took part in a structured low-level exercise program consisting of 30 min slow walking and non-aerobic gymnastics daily. Additionally, physical therapies including hydrotherapy and massage and a validated stress reduction program (Mind/Body Program; Harvard Medical School [16]), were delivered to all patients. No immunosuppressive or antibiotic treatments were allowed throughout the study.

Clinical data and stool samples were collected at admission and at one of the last two hospital days. After 3 months patients were mailed prepaid sample tubes with a letter asking to send the stool samples directly to the analysing laboratory.

### Measurements

#### Bacteriological analyses

Fecal samples were collected from all subjects after defecation. The samples were immediately sent to the laboratory (Enterosan™, Bad Bocklet-Großenbrach, Germany). There, fecal samples were suspended and homogenised in sterile saline. After a serial dilution in saline 0,1 ml of each dilution was surface plated onto a series of selective and nonselective media. The inoculated plates were incubated at 37°C for 1 day under aerobic conditions (for enumeration of Escherichia coli, other enterobacteria, enterococci and other aerobic bacteria), respectively for 3 days under microaerobic conditions (for enumeration of lactobacilli), or for 3 days under anaerobic conditions (for enumeration of bifidobacteria, bacteria of bacteroides-prevotella-porphyromonas-group and clostridia). Plates for enumeration of yeasts and moulds were incubated at 30°C for 3 days under aerobic conditions. Following incubation the colony-forming units were counted. Isolates were identified macroscopically and classified up to the bacterial species by predefined morphological criteria.

#### Analysis of fecal secretory Immunoglobuline A (sIgA)

Measurements of sIgA-were carried out using ELISA-Kits (Immundiagnostik, Bensheim, Germany) according to the manufacturer's instructions. For this, feces were suspended in diluent-buffer. After homogenisation and centrifugation the supernatants were transferred to the microplates, each coated with antibodies specific for sIgA. Anti-sIgA antibody conjugated with peroxidase were used for development. For each well the optical density was measured at 450 nm on a microplate ELISA reader (Dynex, Heidleberg, Germany). The results of the test samples were calculated from the standard curve and expressed as mg/g stool. Stool-ph was measured by standard ph-metry and quantified in 0.5 steps.

### Clinical data

Diagnosis of RA and FM were each established according to the American College in Rheumatology classification criteria of 1987 and 1990 [17,18]. Disease activity of patients with RA was assessed by the Rheuma Activity Index (DAS 28) [19] using quantification of affected and swollen joints, morning stiffness and erythrocyte sedimentation rate (ESR). Activity of FM was assessed by asking the patients for the global severity of the disease-related pain by means of a self-rating 10 cm Visual Analogue Scale (VAS) with a value of 10 indicating maximum pain and 0 indicating no pain. Patients were carefully instructed before first self-ratings on the correct use of the VAS. Subjects height and body weight was measured following a standardised protocol while patients wore light clothing and no shoes after an overnight fast. BMI was calculated as weight (kg)/ height^2 ^(m). Anthropometrical and clinical data were collected by trained study personnel. Dietary habits at baseline were evaluated by a semiquantitative food frequency questionnaire asking by means of 5-point Likert scales for the intake of different food items within the last month with a value of 0 indicating an intake of once or less in the previous month and a value of 5 indicating an intake of at least once daily in this period. The questionnaire asked for the following food items: meat, sausage or meat products, fruits, vegetables or legumes, snacks or fast-food, nuts or almonds and fish. A Mediterranean Diet score was calculated by summarising the values for fruits, vegetables or legumes, nuts-almonds and fish and the reversed values for meat, meat products or sausage and snacks or fast food resulting in a range of 5 to 35 with a value of 35 indicating most favourite nutritional habits. As nearly all patients already used olive oil or canola oil for meal preparations the intake of olive oil was not introduced in the sum score.

### Statistics

Descriptive data are presented as mean ± SD and median with interquartile range, when applicable. For inductive statistical analyses we took all stool parameters to their logarithm, but left the clinical outcomes unchanged, and fitted generalised linear models [20] to the data which included time, treatment group, diagnostic group and baseline values as covariates. The serial correlations were assumed to be exponential in time. All reported tests (group comparisons between fasters and nonfasters as well as between patients with RA and FM) were based on appropriate F-Tests within these models. Correlation analysis was performed with Spearman's correlation coefficient relating the courses of disease activity, stool-ph and sIgA. All statistical computations were performed with SAS/STAT ^® ^statistical software version 9.1 (SAS institute, Cary, North Carolina, USA).

## Results

Baseline characteristics of all 51 patients are presented in table 1. Female patients represented more than 90% of patients with FM and all patients with RA. Pooling the patients according to the received nutritional intervention revealed a significantly higher BMI for fasting patients (27.5 ± 4.8 kg/m^2^; n = 30) compared to non-fasters (mean BMI 24.5 ± 4.0 kg/m^2^; n = 21). In addition, within the RA group fasting patients were older and had more active disease compared to Mediterranean Diet patients. Furthermore, RA patients demonstrated a nonsignificant higher baseline Mediterranean Diet score compared to FM patients (p = 0.145). Regarding the single food items patients with RA had a significant lower intake of sausage and meat products compared to FM patients (p = 0.008). The patient groups differed regarding the prescribed medication at baseline with RA patients showing higher prescription rates of NSAIDs. As expected, disease modifying antirheumatic drugs (DMARDs) and corticosteroids were prescribed in RA patients only.

**Table 1:** Baseline characteristics according to diagnosis and nutritional therapy

	**Fasting**		**Mediterranean Diet**	
	**FM**	**RA**	**FM**	**RA**

N	21	9	14	7
Age, y	52.0 ± 10.0	57.7 ± 6.5	51.6 ± 13.3	49.4 ± 14.3
Female sex (n/%)	19 (90%)	9 (100%)	13 (93%)	7 (100%)
BMI, kg/m^2^	26.9 ± 3.7	28.8 ± 6.9	25.2 ± 3.8	23.2 ± 4.3
Disease Activity				
RA (DAS 28)		62.6 ± 12.3		59.6 ± 12.3
FM (VAS)	5.1 ± 1.5		5.3 ± 1.9	
Medication, n (%)				
NSAIDs	10 (48)	7 (78)	7 (50)	4 (57)
Other Analgetics*	5 (24)	1 (11)	2 (14)	3 (43)
DMARDs	0 (0)	5 (56)	0 (0)	2 (29)
Corticosteroids	0 (0)	3 (33)	0 (0)	3 (43)
Nutritional intake, Mediterranean Diet score (7–35)**	22.3 ± 2.5	24.3 ± 3.5	23.2 ± 3.4	25.9 ± 3.1

Values are mean ± SD if not indicated otherwise. RA, rheumatoid arthritis; FM, fibromyalgia; VAS, Visual analogue scale; BMI = body mass index; NSAIDs, non-steroidal antiinflammatory drugs; DMARDs, disease-modifying antirheumatic drugs. *include opioids, amitryptiline, gabapentin. ** Higher scores indicating better adherence to characteristics of Mediterranean Diet.

### Clinical course

**Figure 1:**
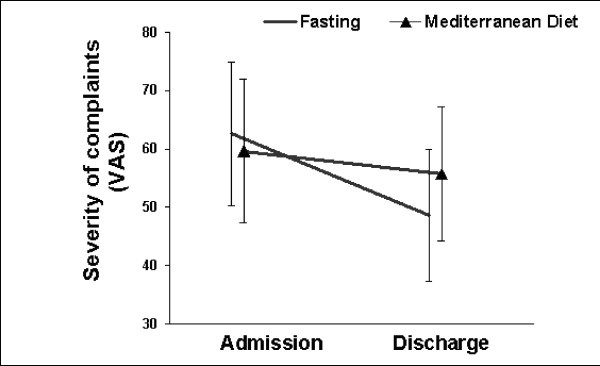
Severity of main complaint (pain) before and at the end of the 2-week treatment period in FM patients (fasting: n = 21; Mediterranean diet: n = 14). *P *= 0.25 for between-group difference of change. Significant within-group difference of change for fasters (*P *= 0.003: t-test), but not for non-fasters (*P *= 0.240).

Adherence with the dietary regimens, as assessed by daily interviews, was excellent. None of the patients discontinued the scheduled treatment program. Mean body weight decreased by -3.3 ± 1.4 kg in fasting patients (RA -3.5 ± 1.6 kg; FM -3.0 ± 1.3 kg) compared to -1.2 ± 0.3 kg in patients on Mediterranean diet (RA -1.3 ± 1.2 kg; FM -1.2 ± 1.8 kg) Fasting was well received by all patients and no relevant adverse events occurred. The severity of disease-related symptoms in FM patients and the disease activity in RA patients were reduced from admission to discharge. In RA patients the clinical improvement tended to be greater in fasting (0.7 score points) vs. non-fasting patients (0.2 score points; group difference 0.5 points, 95%-CI: -0.1 to 1.1; p = 0.09). Fasting FM patients showed nonsignificant greater clinical improvements than non-fasters (group difference 7.8 mm VAS, 95%-CI: -13.4 to 29.1; p = 0.25). Within-group comparisons in both diagnostic groups showed only for fasters a highly significant overall-treatment effect (Fig. 1/2).

**Figure 2:**
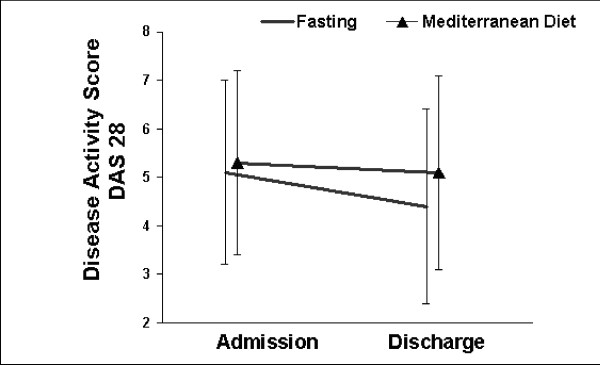
Severity of the Disease Activity Score (DAS) before and at the end of the 2-week treatment period in patients with RA (fasting: n = 9; Mediterranean diet: n = 7). P = 0.09 for between-group difference of change. Significant within-group difference of change for fasters (*P *= 0.007: t-test), but not for non-fasters (*P *= 0.425).

### Stool analysis

Baseline values for all parameters showed large interindividual differences and were statistically comparable between fasters and non-fasters and each diagnostic group. Main data of the microbiological quantification of the aerob fecal flora and of anaerob bacteria and candida during the study course are presented in tables 2 and 3. Stool-ph and concentrations of sIgA are given in table 4. The nutritional interventions were not associated with any significant within-group or between-group (fasters vs. non fasters; FM vs. RA) changes from baseline to the end of the inpatient treatment period for any of these measured parameters. Also, we found no group differences in the composite number of anaerobic species. Three-months-data of stool analysis were available in 43 patients (83%) and did neither reveal differences in any of the stool parameters compared to baseline nor compared to first follow-up. Further quantification of bacteriae and Candida in the pooled fasting or Mediterranean diet groups showed no relevant changes over time. Finally, in correlation analysis the course of disease activity was not related to sIgA concentrations or stool-ph in any of the groups.

**Table 2:** Aerob and microaerophil (LAB) bacterial fecal flora: log. numbers of characteristic bacteria at the three timepoints in the different groups. No statistically significant change between time points within and between groups.

	**Baseline (n = 51)**		**Discharge (n = 47)**		**3 Months-FU (n = 43)**	
	Mean ± SD	Median (IQR)	Mean ± SD	Median (IQR)	Mean ± SD	Median (IQR)

**Escheria coli**						
Fasting, RA.	6.8 ± 1.6	7.6 (6.0–7.9)	7.0 ± 1.6	7.9 (6.0–8.0)	6.1 ± 1.6	6.5 (5.0–7.5)
Fasting, FM	6.7 ± 1.3	7.0 (6.0–8.0)	6.7 ± 1.4	7.0 (6.5–7.7)	6.2 ± 1.4	6.3 (5.2–7.0)
MD, RA	5.6 ± 1.9	5.3 (3.7–7.7)	5.5 ± 2.1	4.8 (3.7–7.7)	6.3 ± 1.8	6.5 (5.3.–7.8)
MD, FM	6.7 ± 1.6	7.5 (5.0–7.8)	6.4 ± 1.6	7.0 (5.0–7.6)	6.2 ± 0.8	6.0 (5.7–6.5)
**Enterococcus**						
Fasting, RA	6.6 ± 1.0	7.0 (6.0–7.3)	6.6 ± 1.3	7.0(5.3–8.0)	6.3 ± 0.7	6.0 (6.0–6.5)
Fasting, FM	6.2 ± 1.1	6.0 (5.5–7.0)	5.6 ± 1.4	5.8(4.3–7.8)	5.9 ± 1.4	6.0 (5.2–7.2)
MD, RA	5.7 ± 0.7	5.5 (5.0–6.0)	6.0 ± 1.3	6.0 (6.0–7.0)	5.7 ± 1.5	5.8 (5.5–6.0)
MD, FM	5.9 ± 1.5	6.2 (5.0–7.0)	6.1 ± 1.5	6.1 (5.2–7.5)	5.8 ± 1.1	5.6 (5.3–6.3)
**Lactobacillus (LAB)**						
Fasting, RA	5.8 ± 0.5	6.0 (5.5–6.3)	5.2 ± 0.8	5.5(5.3–5.6)	5.1 ± 1.0	5.3 (4.3–6.0)
Fasting, FM	5.4 ± 0.7	5.5 (5.3–6.0)	5.3 ± 0.9	5.5(4.7–6.0)	5.3 ± 1.1	5.7 (4.5–6.0)
MD, RA	4.8 ± 0.6	5.0 (4.3–5.3)	5.1 ± 1.0	5.0 (5.0–6.0)	4.2 ± 0.9	4.0 (4.0–4.5)
MD, FM	4.8 ± 1.2	5.0 (4.3–5.7)	5.7 ± 0.7	5.8 (5.5–6.0)	5.2 ± 0.9	5.3 (5.0–6.0)

FU, follow-up; IQR, interquartile range; MD, Mediterranean diet; RA, rheumatoid arthritis; FM, fibromyalgia.

**Table 3:** Anaerob bacterial fecal flora and Candida log. numbers of characteristic bacteria at the three timepoints in the different groups. No statistically significant change between time points within and between groups.

	**Baseline (n = 51)**		**Discharge (n = 47)**		**3 Months-FU (n = 43)**	
	Mean ± SD	Median (IQR)	Mean ± SD	Median (IQR)	Mean ± SD	Median (IQR)

**Clostridia**						
Fasting, RA	5.8 ± 0.4	5.7 (5.7-5.7)	5.7 ± 0.1	5.7 (5.7-5.7)	5.8 ± 0.3	5.7 (5.7-5.7)
Fasting, FM	5.8 ± 0.2	5.7 (5.7-5.7)	5.8 ± 0.2	5.7 (5.7-5.7)	5.7 ± 0.1	5.7 (5.7-5.7)
MD, RA	5.7 ± 0.1	5.7 (5.7-5.7)	5.7 ± 0.2	5.7 (5.7-5.7)	5.9 ± 0.3	5.7 (5.7-5.7)
MD, FM	6.0 ± 0.6	5.8 (5.6–5.9)	5.8 ± 0.1	5.7 (5.7-5.7)	5.8 ± 0.2	5.7 (5.7-5.7)
**Bidifidobacteria**						
Fasting, RA	7.7 ± 1.2	8.0 (7.0–8.5)	7.2 ± 1.1	7.5 (6.6–8.0)	7.7 ± 0.9	7.9 (7.5–8.0)
Fasting, FM	7.6 ± 1.2	7.7 (7.0–9.0)	7.6 ± 1.0	7.5 (7.0–8.2)	8.0 ± 0.9	8.0 (7.4–8.5)
MD, RA	7.5 ± 1.2	8.0 (6.3–8.5)	8.1 ± 0.9	8.2 (7.3–9.0)	7.9 ± 0.9	7.9 (7.3–8.0)
MD, FM	7.5 ± 1.0	7.5 (7.0–8.0)	7.4 ± 1.2	7.4 (6.5–8.2)	7.6 ± 1.4	7.5 (7.0–9.0)
**Candida**						
Fasting, RA	2.2 ± 0.7	1.7 (1.7–2.6)	2.4 ± 0.9	2.0 (1.7–2.8)	2.4 ± 1.1	2.0 (1.7–2.6)
Fasting, FM	2.4 ± 0.9	1.7 (1.7–3.0)	2.6 ± 1.1	2.0 (1.7–2.7)	2.6 ± 1.0	2.5 (1.7–2.5)
MD, RA	2.4 ± 1.0	2.0 (1.7–3.0)	2.1 ± 0.4	1.7 (1.7–2.3)	2.2 ± 0.7	1.7 (1.7–2.6)
MD, FM	2.6 ± 0.8	2.5 (2.0–3.3)	2.4 ± 1.1	1.7 (1.7–3.2)	2.8 ± 1.0	2.8 (2.0–3.3)

FU, follow-up; IQR, interquartile range; MD, Mediterranean diet; RA, rheumatoid arthritis; FM, fibromyalgia.

**Table 4:** Stool pH and secretory immunoglobulin A (sIgA) stool concentrations according to group assignment in the study course: No statistically significant change between different time points within and between groups.

	**Baseline (n = 51)**		**Discharge (n = 47)**		**3 Months-FU (n = 43)**	
	Mean ± SD	Median (IQR)	Mean ± SD	Median (IQR)	Mean ± SD	Median (IQR)

**PH**						
Fasting, RA	7.2 ± 0.8	7.5 (7.0–8.0)	7.5 ± 0.5	7.5 (7.0–7.5)	6.8 ± 0.5	7.0 (6.5–7.0)
Fasting, FM	7.2 ± 0.9	7.0 (7.0–8.0)	7.2 ± 0.8	7.5 (6.5–8.0)	6.8 ± 0.9	6.5 (6.5–7.5)
MD, RA	7.4 ± 0.8	7.0 (6.5–8.0)	7.5 ± 0.5	7.5 (7.0–8.0)	6.9 ± 0.7	7.0 (6.5–7.5)
MD, FM	7.4 ± 0.7	7.5 (6.5–8.0)	7.3 ± 0.8	7.5 (6.5–8.0)	7.2 ± 0.7	7.5 (6.5–7.5)
**IgA**						
Fasting, RA	1.3 ± 1.2	1.2 (0.4–1.4)	1.0 ± 0.7	0.9 (0.6–1.1)	1.8 ± 2.8	0.5 (0.4–1.4)
Fasting, FM	1.1 ± 1.0	0.7 (0.4–1.5)	1.1 ± 0.1	0.7 (0.4–1.7)	1.1 ± 0.9	1.0 (0.3–1.8)
MD, RA	1.3 ± 1.7	0.6 (0.2–2.1)	0.6 ± 0.7	0.4 (0.3–0.6)	0.8 ± 0.5	0.6 (0.5–1.0)
MD, FM	1.0 ± 1.2	0.5 (0.4–0.8)	0.7 ± 0.9	0.5 (0.3–0.6)	0.8 ± 1.1	0.4 (0.3–0.7)

FU, follow-up; IQR, interquartile range; MD, Mediterranean diet; RA, rheumatoid arthritis; FM, fibromyalgia.

## Discussion

It is increasingly believed that the intestinal microflora strongly affects human health, and the quantification of the intestinal bacterial flora quantification as an adjunctive diagnostic method has gained renewed interest in the field of Complementary and Alternative Medicine [11]. Claims of associations between specific commensal bacterial species and health will have to be established in human feeding and intervention studies, but, to date, only a few studies have investigated the influence of diet and fasting on the bacterial flora in patients. We hypothesised that both, Mediterranean diet and fasting would differentially alter the microflora of RA patients and that the clinical course of these patients during the dietary intervention would be connected to changes in the intestinal microflora as assessed by cultural technique and stool sIgA concentration. However, the results of this study do not suggest any relationship between diet, fecal culture analysis, sIgA and disease activity in patients with RA and FM.

Some cross-sectional studies based on cultural techniques have indicated that the protein and fat content of the diet as well as the nature of carbohydrates (simple sugars vs. complex carbohydrates) do affect microflora composition and activity [7,21]. Animal studies support the hypothesis that the intestinal microflora can be modified by diet [8]. In contrast, food restriction has been shown in one study to have little effect on the microflora of rats as measured by conventional anaerobic culture [22], whereas others have observed that food restriction and diet composition both strongly affect the microflora composition as judged by newer diagnostic techniques (denaturing gel gradient electrophoresis) [23]. Generally, data from human clinical studies are limited. Our results contrast to recent findings from a study with 43 patients with RA, which were randomised to an uncooked vegan diet or an ordinary omnivorous diet [24]. After one month, the intervention group showed a significant, diet-induced change in the fecal flora. Furthermore, the clinical improvement was related to the changes in fecal flora, which were assessed by direct stool sample gas-liquid chromatography (GLC). It has also been noted that the GLC method to study overall changes in fecal flora due to a vegan diet might be superior than the classical quantitative culture of stool sample we used [13]. Moreover, the studies' intervention with an uncooked vegan diet rich in lactobacilli clearly differs from the Mediterranean diet that was used in our study. However, in a recent randomised trial on RA patients a Mediterranean diet induced reductions in inflammatory activity and an improvement in physical function to a similar extent as vegan diets in the prior studies [25]. Thus, it may be that vegan diet and Mediterranean diet share nutrients that exert beneficial effects in RA.

Of note, in a randomised trial on fasting and vegetarian diet in RA patients significant differences in the fecal flora were observed between samples obtained at times which coincided with pronounced clinical improvement compared with baseline, versus samples obtained at times of low or no improvement [10]. Again, in this study stool samples were analysed by GLC. Thus, apart from our differing study population with a smaller sample size of RA patients, the conflicting result might be due to a potential higher sensitivity of GLC compared to the classical microbiological cultural analysis used in the present study.

However, we also could not find any effect of the dietary interventions on stool pH and sIgA concentrations (a putative marker of intestinal immune function), which supports the suggestion that Mediterranean diet and fasting do not induce relevant changes in the fecal flora in the short term. To the contrary, in an observational study on patients with chronic pain syndromes (including FM) participation in fasting therapy significantly increased sIgA levels [14]. Yet, baseline sIgA concentrations in this study were markedly lower than in our population, and the increased concentrations at the follow-up corresponded to baseline values in the our study. Therefore, regression to the mean in that study may be a likely explanation for the differing result.

In the present study RA patients tended to show better clinical outcome with fasting than on Mediterranean Diet. However, the interpretation of differences in clinical outcome between the intervention groups is limited by potential baseline differences of nutritional habits. It is known that patients with RA frequently alter their dietary patterns for symptom relief. In fact, In the present study RA patients had a slightly higher nutritional score and a reduced intake of meat compared to FM patients and the whole population showed eating patterns with some aspects of the Mediterranean Diet already realised before study entry. Therefore, the clinical response and changes in fecal flora may have been reduced in the Mediterranean Diet group. Furthermore, due to the non-randomised study design, fasting RA patients were older and had more active disease compared to fasting Mediterranean Diet patients. Thus, we cannot rule out a selection bias in the clinical response to the two types of nutritional interventions.

Another limitation relates to the short intervention time for the Mediterranean diet. Indeed, the clinical benefit of a Mediterranean diet in patients with RA was more evident after 12 than after three or six weeks of intervention time [25]. In contrast, change to an uncooked vegan diet and the return to a Western diet induced alterations in the fecal flora within one to two weeks [13]. Clearly, future studies on the association between Mediterranean diet and intestinal microflora should evaluate longer study periods as minor changes could accumulate over time.

A principal limitation of the present study relates to the small sample size. This implies that no definite conclusions should be drawn from our data. However, the complete absence of effects of the interventions on the bacterial flora makes it unlikely that a larger sample size would have yielded different results. Other factors, as medication, may also have influenced the bacterial flora. Yet, neither probiotics or antibiotics were given, and, within the diagnostic groups there were no other relevant differences of prescribed medication between fasters and patients on Mediterranean Diet. Another limitation that applies to all research testing fecal samples to determine bacterial flora is the influence of the fecal water concentration. This factor may not only vary from individual to individual, but within individuals within the same day. However, drying the stool would not be possible without compromising the presence of certain bacteria.

It may be further argued whether microbiologic analysis of the fecal flora with its limitations in diagnostic accuracy is the appropriate method to assess treatment effects. However, culture is the classical approach for the identification and quantification of bacteria. Most of the data available on the gut bacteria have been generated by cultivation and enumeration [26]. Still, this approach is limited in scope, as a majority of the bacterial species present in feces are not culturable using standard microbiologic techniques. Yet, we believe that the lack of effect of the interventions on sIgA in our study further points to the suggestion that the intestinal microflora is not connected to the dietary treatments and clinical outcome in the selected patient groups. There are now refined developments with molecular analysis tools including the availability of the completed genome sequences [26]. Given their expense, these newer molecular techniques await their broader implementation in clinical research. Clearly, the future utilisation of molecular microflora analysis tools can help to clarify a potential interplay between diet and human microbiota.

Finally, it is important to note that labelling nonpathogenic commensal bacteria as either beneficial or detrimental remains highly speculative. Yet, as we did not found any alterations in the fecal flora of our patients, this issue remains negligible for the interpretation of our results.

## Conclusion

In recent years, the interest of using intestinal flora analysis for clinical purposes has increased. In rheumatic diseases a role for diet-induced alterations in fecal flora has been suggested. The present study revealed that fasting or Mediterranean diet do not affect the bacterial counts and sIgA levels in the intestinal tract in patients with RA and FM in a two-week period and that the clinical outcome of dietary intervention is not related to changes in intestinal flora. Given the numerous claims of associations between the intestinal flora and human health future studies that include newer molecular techniques should clarify the impact of intestinal flora on clinical outcome and the possible modification through diet. Using the classical microbiological technique there seems to be no role for fecal flora analysis in the clinical approach to rheumatic diseases.

Our data further suggest that the efficacy of fasting in the treatment of FM should be addressed in randomised trials, given that the clinical course in both, patients with FM and RA, appeared to be beneficially affected by fasting. For RA the benefit was more apparent, a result which is consistent with data from previous randomised trials which demonstrated fasting to be an efficacious treatment in RA [12,27]. Our data indicate that FM patients might also benefit from fasting treatment. Specific effects of fasting on neuroendocrine regulation, central serotonin availability and quality of sleep have been previously reported [28-30] and point to potential mechanisms of symptom-relief in chronic pain syndromes.

## Competing interests

The author(s) declare that they have no competing interests.

## Authors' contributions

AM conceived the study, the study design, participated in interpretation of data and drafted the article; MR participated in the study design, the data acquisition and helped to draft the article; RL performed the statistical analysis and participated in the interpretation of data; MB participated in the interpretation of data and helped to draft the article; JL participated in the data acquisition and helped to draft the article; MS participated in the interpretation of the data and the conception of the study; GD contributed to the conception of the study, to the interpretation of data and participated in drafting of the article. All authors have read and approved the final manuscript.

## Pre-publication history

The pre-publication history for this paper can be accessed here:


